# Nanoparticles for Signaling in Biodiagnosis and Treatment of Infectious Diseases

**DOI:** 10.3390/ijms19061627

**Published:** 2018-05-31

**Authors:** Clara I. Colino, Carmen Gutiérrez Millán, José M. Lanao

**Affiliations:** 1 Area of Pharmacy and Pharmaceutical Technology, Department of Pharmaceutical Sciences, University of Salamanca, 37007 Salamanca, Spain; ganda@usal.es (C.I.C.); carmengutierrez@usal.es (C.G.M.); 2 The Institute for Biomedical Research of Salamanca, 37007 Salamanca, Spain

**Keywords:** nanoparticles, signaling, bacterial infections, viral infections, quorum sensing, point-of-care testing

## Abstract

Advances in nanoparticle-based systems constitute a promising research area with important implications for the treatment of bacterial infections, especially against multidrug resistant strains and bacterial biofilms. Nanosystems may be useful for the diagnosis and treatment of viral and fungal infections. Commercial diagnostic tests based on nanosystems are currently available. Different methodologies based on nanoparticles (NPs) have been developed to detect specific agents or to distinguish between Gram-positive and Gram-negative microorganisms. Also, biosensors based on nanoparticles have been applied in viral detection to improve available analytical techniques. Several point-of-care (POC) assays have been proposed that can offer results faster, easier and at lower cost than conventional techniques and can even be used in remote regions for viral diagnosis. Nanoparticles functionalized with specific molecules may modulate pharmacokinetic targeting recognition and increase anti-infective efficacy. Quorum sensing is a stimuli-response chemical communication process correlated with population density that bacteria use to regulate biofilm formation. Disabling it is an emerging approach for combating its pathogenicity. Natural or synthetic inhibitors may act as antibiofilm agents and be useful for treating multi-drug resistant bacteria. Nanostructured materials that interfere with signal molecules involved in biofilm growth have been developed for the control of infections associated with biofilm-associated infections.

## 1. Introduction

Common infectious diseases have been reasonably well controlled by a broad range of anti-infective agents, but recently, due to the massive use of these drugs, new resistant strains have appeared. This fact and the interest in improving existing therapies compel the scientific community to keep searching for new ways to combat infections [1]. In this area, nanotechnology has a variety of practical roles to play, from drug delivery to diagnostics. Nanoparticulate systems offer a versatile platform that can offer different intrinsic properties depending on the nature of the nanoparticle. Moreover, they can be functionalized, which broadens their potential and opens new possibilities for customizing them according to the intended aim [2]. 

Nanoparticles (NPs) can include a wide variety of molecules, offering a multifunctional platform for drug targeting, diagnostic techniques and even theranostics that have already demonstrated their potential in clinical trials [1,3,4,5].

Advances in nanoparticle-based systems for the diagnosis and treatment of infectious diseases constitute a promising area of research with important implications for the treatment of bacterial infections, especially in the fight against multidrug resistant strains and bacterial biofilms. In addition, nanosystems may be useful in the diagnosis and treatment of viral infections such as HIV and hepatitis, the treatment of fungal infections, or as vaccines [6,7].

Nanoparticles functionalized with specific molecules such as proteins, antibodies or DNA may modulate pharmacokinetics and targeting recognition and increase the efficacy of anti-infective drugs. The characteristics of certain types of nanoparticles and additional functionalization give them the ideal properties for application in diagnostic analysis, even allowing for the miniaturization and simplification of some traditional techniques of pathogen detection [1].

In addition, nanoparticles constitute an interesting platform for theranostic applications. For instance, systems that combine drugs and certain kinds of nanoparticles allow therapeutic agent delivery as well as the imaging of a target organ or tissue [4,8,9].

An interesting use of nanoparticles is in the design of biosensors: analytical devices that incorporate biological material, biologically derived material or a biomimic with a physicochemical transducer or a transducing microsystem [10].

Biosensors could allow for the measurement of a specific analyte or group of analytes that can be detected through a receptor fixed onto the surface of a transducer that detects the signal produced by the analyte recognition. Although this concept was initially developed for cancer biomarkers [10,11], if an appropriate biomarker were identified, it could be applied similarly to other pathological conditions [12,13,14]. 

Nanoparticles offer a platform in which different kinds of proteins and peptides such as specific ligands or enzymes can be included [15,16,17,18] and allow the identification and/or quantification of molecules of interest, even in multiplex detection.

Additionally, very sophisticated multilayered nanoparticles could be designed to include targeting molecules, as both biosensors for detecting surrogate molecules expected in infected cells prior to the delivery of a nanoparticle cargo, and moreover, as a feedback control mechanism for the amount delivered [19].

Quorum sensing is a stimuli-response chemical communication process correlated with population density that bacteria use to regulate biofilm formation. The disabling of quorum sensing is an emerging approach for combating its pathogenicity. Natural or synthetic quorum-sensing inhibitors may act as anti-biofilm agents and be useful in treating multi-drug resistant bacteria.

Nanostructured materials which interfere with the signal molecules involved in biofilm growth have been developed for the control of biofilm-associated infections.

The aim of this review is to show the most recent advances in the role played by molecular signaling via nanoparticles and nanomaterials in the diagnosis and treatment of infectious diseases.

## 2. Nanoparticles for Bacterial Detection

Traditional methods used for the identification of bacteria are based on the culture of the microorganisms on agar plates and the characterization of their phenotypic properties. These techniques are laborious, slow and unspecific in some cases. New methods for the identification of bacteria based on the principles of molecular biology have also been developed and specially polymerase chain reactions (PCR) based methods [20,21]. In the last decade, different methods based on the combination of sensitive techniques such as surface-enhanced Raman spectroscopy (SERS), electrochemical methods or fluorescence methods combined with the use of nanoparticles, especially metal nanoparticles, for rapid and sensitive identification of bacteria have been developed. In addition, some of these methods also allow the killing of the bacteria. These methods have some general advantages in comparison with traditional methods such as shorter analysis times and the possibility to identify different types of bacteria simultaneously.

Different commercial tests based on nanosystems using biochemical, immunological and bioluminescence procedures are currently available for detecting pathogenic agents. Methodologies based on gold or silver nanoparticles, glass nanospheres or quantum dots among others, have been developed to detect specific agents or to distinguish between Gram-positive and Gram-negative microorganisms.

Rapid and sensitive detection of pathogenic bacteria is an important research area considering its implications for healthcare, the environment, food, etc. [6].

Traditionally, different physico-chemical and immunological methods have been developed for bacterial detection such as fluorescence spectroscopy, mass spectrometry, enzyme-linked immunosorbent assay, etc. [22].

The detection of bacteria using biosensors is not a new procedure [23,24]. However, in recent years, important advances have been made in the development of biosensors based on immunological methods that take advantage of the optical and electrical properties of nanoparticles, particularly metal nanoparticles [25]. The combination of sensitive techniques such as magnetic sensors, Surface-enhanced Raman Spectroscopy (SERS), electrochemical methods and fluorescence methods with different types of nanoparticles such as gold, silver, magnetic, silica and functionalized nanoparticles, among others, has allowed the development of very specific and sensitive methods for the diagnosis and killing of bacteria, with numerous applications both in biomedicine and in other fields. The main applications of these methods are the detection of multi-resistant bacteria in healthcare and veterinary practice and in the food industry.

### 2.1. Magnetic Sensors

Magnetic nanoparticles labeled with antibodies may be used for bacterial detection. The nanoparticle-antibodies conjugate, attach to the bacteria, and then the nanoparticles are removed using an external magnetic field. Then, different methods of detection and quantification of bacteria, such as scanning electron microscopy (SEM), transmission electron microscopy (TEM), fluorescence microscopy etc., may be used [6,26].

Tuberculosis is a major health problem worldwide, especially in undeveloped countries, and about 3 million people are estimated to remain undiagnosed or untreated. In addition, *Mycobacterium tuberculosis* strains are associated with higher morbidity and mortality in infected patients, making their prevention, diagnosis and cure crucial [27,28]. 

A magnetoresistive biosensor to detect *Mycobacterium bovis* (BCG) bacteria for tuberculosis diagnosis based on the use of magnetic nanoparticles has recently been developed [29]. This system uses a magnetoresistive biochip whose surface is functionalized with specific BCG antibodies. When magnetic nanoparticles reach the surface of a biochip a change in magnetoresistance is created by the magnetic field and detected by the sensors [29]. This sensitive method is an alternative to classical methods of detecting of tuberculosis such as the Ziehl–Neelsen sputum smear microscopy image test for tuberculosis detection, which has a poor sensitivity.

In parallel, a magnetophoretic immunoassay sensor for early diagnosis of tuberculosis in the culture supernatants from sputum samples has been developed. Antigens of *Mycobacterium tuberculosis* such as culture filtrate protein (CFP)-10 may be detected by this method. The system uses two different kinds of nanoparticles: gold nanoparticles for signaling and magnetic nanoparticles for separation with two kinds of monoclonal antibodies [30].

### 2.2. Surface Enhanced Raman Spectroscopy (SERS)

Raman spectroscopy is a technique which provides information about the molecular vibrations of molecules or groups of atoms when a monochromatic light is used. When laser light irradiates a sample, scattered light is detected; this phenomenon is known as the Raman effect and a spectrum of the sample is obtained by plotting the intensity of the scattered light against frequency. Raman spectroscopy provides detailed specific sample characterization of the material analyzed and is widely used for material identification.

Surface-enhanced Raman Spectroscopy (SERS) is a method that amplifies Raman signals using nanoscale metal surfaces such as gold or silver among other metals. These metallic nanosystems are called plasmonic nanoparticles and exhibit specific optical properties when exposed to laser light, increasing the method’s sensitivity. Surface plasmons are electromagnetic waves that propagate on the surface of a conductor. Surface plasmon resonance (SPR) is a phenomenon associated with this kind of nanoparticle that arises from the interaction between the surface plasmon of a nanoparticle and an incident electromagnetic wave (e.g., laser), producing a quantum effect due to a change in its electronic structure, which depends on the characteristics of the nanoparticle [31,32].

SERS is a powerful method for the identification of certain substances or organisms with some limitations, such as interference related to the nature of the matrix [32]. An important application of SERS is molecular detection and, more specifically, the detection of live bacteria, which has implications for healthcare and the food industry [33,34].

Currently, the usual method for the detection of bacteremia is blood culture; however, in recent years, different bacterial identification methods based on the use of SERS combined with metallic nanoparticles have been proposed. They may be subdivided into label and label-free SERS methods [34].

#### 2.2.1. Label SERS

Label SERS is based on the use of SERS tags, which are entities incorporating metallic nanoparticles that can be employed to label a target molecule and indirectly identify it by using SERS methods. Plasmonic nanoparticles, mainly gold nanoparticles, may be used to synthetize SERS tags [35]. In addition, SERS tags may be functionalized using recognition molecules such as monoclonal antibodies, aptamers or small molecules to increase specificity [34,36]. SERS tags are useful in biomedical imaging with potential application in cancer therapeutics and infectious diseases detection [34,37].

Label SERS functionalized with monoclonal antibodies incorporating different kinds of nanoparticles, such as silver or gold, have been proposed for the detection of different types of bacteria, such as *Salmonella* and *Staphylococcus aureus* among others. Monoclonal antibodies with a high recognition capacity to target a specific surface protein of bacteria are used [22,38,39].

Hybrid systems composed of gold nanoparticles conjugated with Rhodamine 6G (Rh6G) modified monoclonal AC04 antibodies combined with single-walled carbon nanotubes (SWCNT) with SERS detection have been developed for the selective detection and killing of multi-drug resistant *Salmonella typhimurium*. This nanohybrid system provides a significant enhancement of the Raman signal, increasing the method’s sensitivity. In addition, the near-infrared (NIR) irradiation of this nanohybrid system produces bacterial death and selectively eradicates 99% of the bacteria [38]. Also, nanoprobes prepared by the immobilization of a specific monoclonal antibody onto the surface of nanoparticle beds, a microfluidic electrophoresis device and on-line SERS have been developed for the rapid detection of *Salmonella enterica* and *Neisseria lactamica* with high sensitivity and specificity [22].

#### 2.2.2. Label-Free SERS

In recent years, label-free methods for bacterial detection have been developed. One rapid label-free detection method for bacteria in blood is based on the combination of a universal sample preparation process using 10 mL of whole human blood treated with a lysis buffer and the use of a bacterial concentrator to concentrate the microorganisms, producing an enriched sample of viable microorganisms for downstream analysis by SERS. An aliquot is placed on a SERS chip containing a SiO_2_ substrate coated with gold nanoparticles. This method allows for the detection of microorganisms such as *S. aureus* or *Escherichia* among others with a very good specificity and sensitivity and hence with important implications for clinical practice [40,41].

Another rapid real-time analysis based on a label-free SERS biosensor that uses the in situ synthesis of silver nanoparticles has been developed for the identification of Gram-positive and Gram-negative bacteria [42]. In addition, one specific label-free SERS method permits the identification of *Klebsiella pneumoniae* resistant strains. In this method, an aluminosilicate substrate with a 50 nm gold layer was used in a drop coated deposition SERS method. The method may be affected by the heterogeneity of the sample and a multivariate statistical method was used for rapid subtyping of resistant strains [43].

*Pseudomonas aeruginosa* is a common bacteria in airway infections associated with patients with cystic fibrosis. Hydrogen cyanide (HCN) is a *P. aeruginosa* biomarker with a specific signal in the Raman spectrum. The use of highly sensitive gold-coated silicon nanopillar SERS substrate allows for the enhancement of the Raman signal via the formation of electromagnetic “hot spots”. This method allows for the detection of the biomarker HCN from clinical *P. aeruginosa* isolates [44].

### 2.3. Plasmonic Nanocavities

Localized surface plasmons (LSP) are a phenomenon generated by light when it interacts with conductive NPs that are smaller than the incident wavelength. Nanostructures with LSP resonances are used to enhance electric signals near the nanostructure. A single nanohole in a metal layer is capable of supporting a LSP [45]. New technologies based on nanocavity-shaped photonic crystals with strong plasmonic signals have been developed with potential applications in bacterial detection [46,47,48].

This method involves the production of quasi-periodic plasmonic nanocavities by patterning a Thue–Morse (T–M) array of nanoholes in a polymeric film to obtain metallic gold nanocavities that allow the propagation of surface plasmons [46,47]. Surface plasmonic resonances of metallic nanostructures can be either suppressed or enhanced depending on their geometric size [49]. These kinds of structures, combined with SERS, permit rapid and highly sensitive bacteriophage detection of pathogenic bacteria such as *Brucella* sp. [48].

### 2.4. Electrochemical Biosensors

Electrochemical affinity biosensors are based on immunoassays methodology combined with electrochemical detection. Electrochemical detection is a very sensitive method which uses the electrical current that is produced from oxidation or reduction reactions associated with biological processes. These methods may be used for bacterial detection of molecular biomarkers from different pathogenic infections [50,51,52].

*Escherichia coli* O157:H7 is a bacteria associated with food poisoning via the release of a toxin called Shiga that causes diarrhea and severe damage to the kidneys. Biosensors based on gold nanoparticles combined with polyclonal antibodies have been developed. A complex formed by magnetic nanoparticles conjugated with monoclonal antibodies was used for the separation of *E. coli* from the solution. Then the magnetic complex was used for the electrochemical detection of *E. coli* bacteria [53]. 

**Figure 1:**
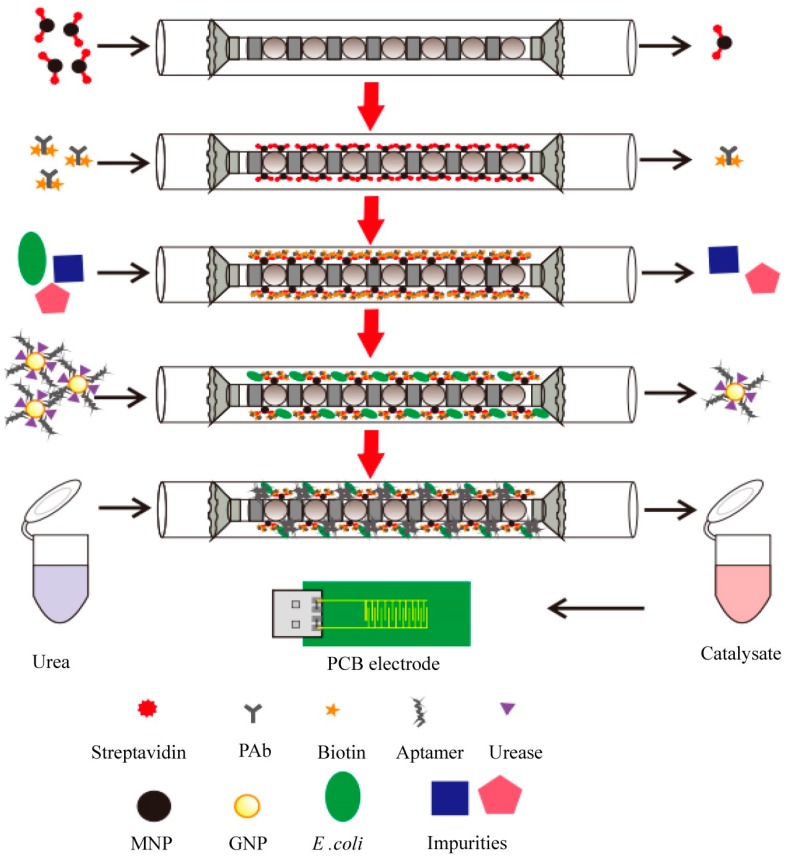
Electrochemical aptasensor for the detection of *E. coli* O157:H7 [54]. Reproduced with permission.

More recently, another group has been working on an electrochemical aptasensor that uses coaxial capillary with immune magnetic nanoparticles for the separation of bacteria [54]. Streptavidin-modified magnetic nanoparticles (MNP) are injected into the coaxial capillary and are subjected to a magnetic field. Biotinylated polyclonal antibodies (PAbs) are injected to produce immune magnetic nanoparticles and a sample containing bacteria is injected into the capillary to form a MNP-PAb bacteria complex called “magnetic bacteria”. Gold nanoparticles (AuNP) modified with the aptamer against the bacteria and urease are injected into the system to produce a MNP-PAb-bacteria-aptamer-GNP-urease complex called “enzymatic bacteria”. Urea is injected into the capillary and then ammonium and carbonate ions are produced by the catalysis of the urea by the bacteria. This produces a reduction in the impedance that is measured by the PCB gold electrode and is related to the concentration of the bacteria. This method is very rapid and sensitive for the detection of *E. coli* O157:H7 (Figure 1) [54].

Nanodecorated electrodes have also recently been developed for the detection of bacteria such as *E. coli* and *Streptococcus pneumoniae* [55,56]. 

This methodology is based on the adherence of metallic nanoparticles to the bacteria’s surface after which nano-decorated bacteria may be detected through electrochemical detection [56]. Silver nanoparticles may interact with the surface of Gram-negative bacteria such as *E. coli*, especially in the range of 1–10 nm, disturbing its functioning and producing a bactericidal effect [57]. Using this principle, *E. coli* bacteria were decorated with silver nanoparticles around 10 nm in diameter due to the high affinity of silver nanoparticles to the surface of the bacteria. The chronoamperometric response of the silver nanoparticles was measured using a carbon fiber electrode. The *E. coli* concentration on the sample may be correlated with the amperometric response [56].

In the same way, detection of pneumococcus such as *S. pneumoniae* has been carried out using electrical methods by measuring the change in electrical properties such as conductance of nanodecorated bacteria. In this case, the bacteria are captured by the pneumococcal C-polysaccharide (PnC) antibody and then gold nanoparticles are conjugated with the PnC antibodies on the surface of the bacteria. By using interdigitated electrodes, the conductance change is correlated with the bacteria concentration. *S. pneumoniae* concentrations of about 10 CFU/ mL may be detected with this method [55].

**Figure 2:**
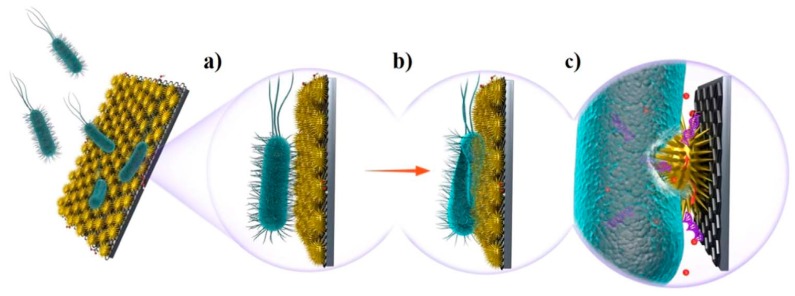
Scheme of the capture by the binding affinity of the electrode sensor and the *E. coli* bacteria (**a**), nano-piercing process on the bacterial wall (**b**) membrane damage, (**c**) cytoplasm leakage and killing of the bacteria [58]. Reproduced with permission.

An alternative is to use nanodecorated impedance electrode sensors to integrate the capture, killing and sensitive detection of the bacteria into a multi-function system. This electrode sensor was manufactured using Zn-CuO nanoparticles and Graphene oxide nanosheets on a nickel porous electrode. With this kind of electrode, capture and killing of bacteria may be realized as seen in Figure 2. In addition, a high detection sensitivity of about 10 CFU/mL of *E. coli* in blood samples was obtained [58]. 

### 2.5. Fluorescence Methods

Fluorescence immunoassay is a sensitive technique that can be used in the measurement of many substances, mainly proteins, and in the quantification of antigens from viruses or bacteria. Fluorescein isothiocyanate (FITC) is a common fluorescent label that produces conjugates with proteins and other biological materials with applications in imaging and sensitive analysis. Different kinds of fluorescent nanoparticle conjugates may be used for bacterial detection.

Immunoassay using fluorescent antibody-nanoparticle conjugates may be applied for the detection of Gram-negative bacteria such as *E. coli* or *P. aeruginosa*. Conjugates of functionalized silica nanoparticles with FITC allow for the rapid detection of *Escherichia coli* O157:H7 by flow cytometry in beef samples. Rabbit anti-*E. coli* O157:H7 antibody was used to recognize *E. coli* O157:H7. Protein A, a stable protein extracted from *S. aureus*, was used for antibody coupling on silica nanoparticle surfaces. The method is rapid, selective and sensitive for the detection of *E. coli* [59].

Immunoassay methodology using small molecules as a substitute for antibodies with a strong affinity to the bacteria, such as certain antibiotics, have been proposed. Vancomycin is a glycopeptide antibiotic with affinity to gram-positive bacteria due to the multivalent hydrogen binding effect. Mesoporous silica nanoparticles modified with vancomycin have been developed for the recognition and killing of Gram-positive bacteria. Mesopores of vancomycin silica nanoparticles were loaded with a fluorescent molecule (FITC). After incubation with FITC and bacteria, the detection was performed by measuring the fluorescence with high specificity and sensitivity. With this method, the detection and killing of Gram-positive bacteria such as *S. aureus* was performed [60].

Also, glucose stabilized silver nanoparticles labeled with a pyrimidine fluorescent derivative were attached to the monoclonal antibody (IgG) of *Pseudomonas aeruginosa*. The fluorescence response was able to be enhanced when the fluorophore was localized near a metal surface due to the characteristics of the metallic nanoparticle. This nanosystem allows for the detection of bacteria in water, agriculture samples, soil, and different types of food [61,62].

CdSe/ZnS quantum dots incorporated into SiO_2_ microspheres constitute a fluorescent labeled system for the detection of *S. typhimurium*. The conjugate between fluorescent nanoparticles and bacteria may be detected by fluorescence microscopy. The detection limit was 3.3 × 10^2^ CFU/mL, and this method may be applied to the detection of other bacteria [63].

Bacterial detection and killing in liquid- and solid-phase systems can also be achieved using surface-coatable NIR response fluorescent nanoparticles. Polydopamine (PdA) is a biomaterial with adhesive properties. Carbonized polydopamine (FDA) may be functionalized with polyethylenimine (PEI) to form fluorescent carbon nanoparticles (FDA:PEI). The cationic surface of these nanoparticles may bind to the anionic bacterial wall of Gram-positive and Gram-negative bacteria such as *S. aureus* or *E. coli* through changes in the fluorescent signal. In addition NIR irradiation of carbonized nanoparticles produces heat and kills the bacteria [64].

Food poisoning produced by bacteria is an important cause of disease and death. The bacteria that cause the most illnesses and deaths are *Salmonella enterica*, *E. coli* and *Clostridium* sp.

Janus nanoparticles are colloidal systems with two spatial domains that have different physicochemical properties. The use of Janus micromotors as mobile sensors has been proposed for the detection of toxins released by enterobacteria. Magnetocatalytic Janus micromotors encapsulating phenylboronic acid (PABA) modified graphene quantum dots based on platinum nanoparticles that produce blue fluorescence emission allow the fast monitoring of enterobacterial contamination in food [65,66].

### 2.6. Fluorescence Resonance Energy Transfer (FRET)

Fluorescence resonance energy transfer (FRET) is a mechanism describing energy transfer between two light-sensitive substances. A donor chromophore, in its electrically excited state due to the excitation of incident light, may transfer energy to an acceptor chromophore. This methodology permits the evaluation of the distance between two chromophores to within several nanometers. A limitation of FRET is that this transfer process is effective only when the distance separating two molecules is smaller than 10 nanometers. FRET allows for the measurement of the interaction between biological molecules within nanoscale [67].

An interesting application of this technique is the study of molecular interactions inside living cells with improved resolution and sensitivity, with potential applications on pathogen-induced host receptor signaling based on protein–protein interactions related with signaling events in infected host cells [68,69].

FRET has recently been proposed for the detection and quantification of *Mycobacterium tuberculosis* DNA using gold nanoparticles and fluorometric detection [70].

## 3. Nanoparticles for Viral Detection

Different kinds of nanoparticles have demonstrated their potential for the diagnosis, treatment and prevention of viral infections in many applications, especially those nanoparticles with viral material or systems that mimic virus characteristics. This section focuses on the diagnostic techniques developed for the identification and/or quantification of viruses themselves, and more specifically of the viruses that have been more thoroughly investigated and have more relevance [71,72,73,74,75,76,77].

It is interesting to remark that in therapeutics against viruses, some specific kinds of nanoparticles have arisen: the so-called “virus-like particles” (VLPs) [78]. They are nanoparticles formed from viral proteins that assemble in structures similar to authentic virus particles although they lack infectious nucleic acid sequences. VLPs can be used for the detection of pathogens, as therapeutic agent carriers and as scaffolds for vaccines against bacterial/viral pathogens [79].

They have been thoroughly investigated in the field of contrast agents, and have been observed to harness the intrinsic properties inherited from their original virus, such as the natural interaction of viruses with certain cells. Moreover, VLPs can also be customized with a variety of biomolecules which significantly broadens their potential uses and advantageous properties [15,18,80].

As previously stated for bacterial detection, the addition of nanoparticles, to common diagnostic techniques, usually improves their characteristics, leading to a higher sensitivity of detection systems in some cases allowing for the detection of even femtomolar amounts of virus [81]. Early diagnosis is always desirable for the control of infectious diseases. Different strategies have been implemented using nanoparticles to improve analytical technique characteristics, even allowing the development of simple and fast point-of-care (POC) assays to diagnose in situ in remote regions.

Although devices based on nanoparticles could imply higher production costs in some cases, other approaches such as the nanoparticle-based immunochromatographic strips, can be easily and cheaply scaled to mass production.

On the other hand, even for some techniques reviewed here that have a similar sensitivity to the usual conventional techniques used for virus diagnosis, the application of nanoparticles offers the key advantage of resulting in diagnostic tests that can be applied in these remote locations with very basic equipment and a lack of specialist trained personnel. The unique and versatile properties of nanoparticles themselves and the molecules that can be associated with them enable fast, sensitive and cost effective diagnoses [82]. 

### 3.1. Magnetic Nanoparticles

As previously mentioned for bacterial detection, magnetic nanoparticles have also been used for the separation of viruses associated with them, which can lead to an enrichment of the sample in the component of interest and a consequent enhancement of detection sensitivity. In addition, magnetic nanoparticles can also be implemented in biosensors as reporter labels that allow magnetoelectronic viral detection [83]. 

Nanoparticles from different magnetic iron oxides present superparamagnetism, which allows the sorting of such superparamagnetic iron oxide nanoparticles (SPIONs) by exposure to magnetic fields. Magnetic-activated cell sorting (MACS) with antibody-conjugated magnetic particles has been coupled to other signals for specific antigen detection [84].

Functionalized magnetic NPs have been applied in different diagnostic techniques to detect hepatitis antigens [84,85,86] or DNA in combination with PCR amplification [87] among other possibilities. 

Different portable biosensors based on magnetic nanoparticles have been developed for rapid virus detection. Electrically active magnetic (EAM) polyaniline nanostructures have been used for Influenza A virus due to their association with antibodies against the surface glycoprotein hemagglutinin (HA). Making polyaniline electrically active by the addition of a protic solvent allows these structures also to be used as the electrical transducer of a rapid, specific and sensitive electrical biosensor [88].

With a similar aim, magnetic nanobeads coated with specific antibodies and combined with a microfluidic chip with an interdigitated array microelectrode have been implemented in a biosensor able to quantify the influenza virus on the basis of the impedance measured for the complexes formed with viruses present [89]. This system showed sensitivity and specificity similar to the usual PCR-based diagnostic method.

Another method, based on magnetizable nanoparticles and electrochemical detection has been designed for the detection of *E6-HPV16* gene, which has been demonstrated to be implicated in mechanisms of human papilloma virus (HPV)-related carcinogenesis. Its combination with PCR improved DNA isolation in comparison with standard PCR, allowing detection of a lower oncogene level [90].

### 3.2. SERS

As has been previously explained, the application of nanoparticles to Raman spectroscopy amplifies the signals, leading to SERS that has been applied for the detection of different kinds of virus.

For hepatitis detection, a highly selective and sensitive immunoassay has been developed with a SERS-active substrate incorporated into a microfluidic device. The formation of a sandwich structure of fuchsin-labeled immuno-Au nanoflowers with HBsAg and the antibody immobilized on the SERS-active substrate based on Au–Ag coated GaN allows for virus detection with low detection limits [91].

The nanoplasmonic properties of gold nanoparticles have been applied in human immunodeficiency virus (HIV) load quantification from whole blood samples. Thus, a detection platform with highly specific antiviral antibodies fixed to the biosensing surface has been demonstrated to be able to detect and quantify multiple HIV subtypes and could also be adapted for the detection of other pathogens that have well-described biomarkers [92].

Variations of the methods based on these inherent properties of gold nanoparticles have been applied in respiratory virus, even allowing for the distinction among different influenza viruses and hepatitis viruses [93,94,95,96]. An optofluidic-nanoplasmonic sensor that could be used as a POC Ebola analysis, even in biodefense contexts, has been designed. This nanohole based sensing platform has demonstrated its ability to detect intact viruses from biologically relevant media with an easy sample preparation and the authors suggest that it could be extrapolated to other viruses [97].

### 3.3. Electrochemical Biosensors

Due to their own nature, metallic nanoparticles such as gold nanoparticles have been widely used in electrochemical detection [98].

Different biosensors based on nanoparticles have been applied in influenza virus detection, among others [99,100], since they have ideal characteristics for designing biosensors for POC assays that can offer diagnostic results faster, easier, at lower cost than conventional techniques and with good selectivity and sensitivity.

Electrochemical biosensors based on gold nanoparticles demonstrated their ultrasensitivity in detecting single-stranded DNA (ssDNA) of HPV and also hepatitis B virus (HBV) [101]. An electrode integrating graphene and polyaniline nanowires has also been proposed as a way to improve its DNA detection sensitivity [102].

On the other hand, immunoassays based on complementary metallicoxide semiconductor (CMOS) image-sensor technology using an indium nanoparticle (InNP) substrates, have been used for hepatitis virus detection [103].

In the research field of Ebola diagnosis, experts have expressly pointed to the need to develop a biosensor that allows the detection of Ebola viruses at the point of care. By analogy with the application of nanoparticles to diagnostic techniques developed for other viruses and on the basis that miniaturized chips with immobilized antibodies have demonstrated their capacity to detect pM levels of other biomarkers [104,105], some authors propose that the miniaturization of electrochemical immune-sensing methodology would be a feasible means to develop devices for rapid and in situ Ebola screening [106].

### 3.4. Fluorescence-Based Methods

The use of fluorescent nanoconstructs as probes has been proposed for the improvement of virus detection techniques based on immunoassays [107,108]. Some authors have developed a fluorescent diagnostic device compatible with a smartphone camera that transmits its data to a central data base system, thus proposing an interesting POC testing method for influenza screening that could even result in an international real-time surveillance system [109].

Some authors have also combined metallic nanoparticles with fluorescence enzyme-linked immunosorbent assay (FELISA) for hepatitis B diagnosis. Thus, by employing dually labeled gold nanoparticles, an enhancement in HBsAg detection limits of conventional ELISA has been observed [110].

Gold nanoparticles and quantum dots have been used for virus detection by FRET, as electron acceptor or donor respectively in FRET pairs. The fluorophore conjugated to specific antibodies undergoes a conformational change when binding to the virus, which changes the distance between the fluorescent molecules or the fluorescent/quencher molecules and a measurable change in the resonance energy transfer is observed. They have been applied to Porcine Reproductive and Respiratory Syndrome Virus (PRRSV) but could be used for other viruses by including the appropriate antibody [111].

Gold and silver nanowires have also been applied as quenchers to immobilize Molecular Beacon hairpin [94]. This structure, consisting of looped strands of DNA with a fluorophore and a fluorescence quencher at the opposite terminus, takes on its open bright state in the presence of the specific target strand of DNA or RNA. Nanowire combinations such as nanobarcodes could accomplish multiplexed assays by designing the oligonucleotides of different nanowires to be complementary to target DNA sequences of different viruses, as has been demonstrated for the simultaneous detection of hepatitis A virus, hepatitis C virus, West Nile Virus, HIV and severe acute respiratory syndrome virus [112].

Quantum dots (QD) have also been explored just as a fluorophore. They allow the detection and monitoring of viral infection through the detection of bioconjugates obtained due to antibody affinity or other interactions. Using different colored QDs could even provide additional information simultaneously, such as structural changes in cytoskeleton. They have demonstrated better sensitivity than respiratory syncytial virus (RSV) detection based on antibodies conjugated to fluorescein isothiocyanate (FITC) [94]. They have also demonstrated their potential for influenza virus detection in clinical samples without any pretreatment [113] and for early stage diagnosis of HIV infections [114].

QDs have also been suggested for hepatitis detection but no better sensitivity than other methods has been proved for this virus [73,115].

### 3.5. Other Optical Biosensing Methods

Colorimetric detection has been used in the detection of respiratory viruses. An enhanced plasmonic ELISA was developed for RSV detection by combining alkaline phosphatase-mediated AuNPs-based colorimetric assay with metal ion sensitized dephosphorylation of ATP [116].

Regarding influenza virus, alkaline phosphatase (ALP) has been used as a signal tag for immunoreaction. Color change was observed in the presence of the virus due to silver deposition on the surface of gold nanoparticles induced by the enzyme. Coupled with magnetic enrichment, this method has been demonstrated to be simple, fast and highly sensitive, allowing H9N2 virus detection directly in complex samples [117].

Metallic nanoparticles have been demonstrated to improve HIV-1 p24 antigen assay. Nanoarrays obtained by nanolithography have shown an enhancement in the detection limit as well as faster detection than the conventional colorimetric enzyme-linked immunosorbent assay (ELISA) [75]. A microchip based on microfluidics and nanoparticles has been developed which allows diagnosis on-site of both HIV and syphilis. This device replicates the ELISA steps and allows the detection of antibody markers within scarcely few minutes at low cost, with enough sensitivity and specificity and requiring just a few microliters of blood sample. Thus, this mChip miniaturizes ELISA techniques and, implemented in a handheld instrument easy to operate, provides a fast and affordable assay that can be used in the most remote regions [118].

Dual-luminophore-doped silica nanoparticles with different surface modifications have been studied for multiplexed analysis. In combination with flow cytometry, it has been suggested that these systems have interesting advantageous properties in the detection of pathogens, especially for those that have problems with normal dyes due to their minimal specific antigens. Results revealed that these nanoparticles have high signal amplification, excellent photostability and easy surface bioconjugation for biomarker detection, which highlights this system as an ideal biolabeling reagent in antigens and nucleic acids detection [119].

Other methods based on colorimetric detection have been applied to virus detection. Through association of gold nanoparticles with reverse transcription loop-mediated isothermal amplification, a very simple assay for hepatitis E was developed, whose results can be evaluated with the naked eye due to color changes. It has been proposed as an alternative to other expensive and time-consuming methods usually employed [120]. Fe_3_O_4_ magnetic nanoparticles have been also applied as nanozyme probes, harnessing their natural intrinsic peroxidase-like activity that can be visually detected due to the obvious color reaction. By labelling them with specific antibodies and in the presence of peroxide substrates, they have been studied for immunomagnetic Ebola virus detection [121].

Other DNA detection methods based on chemiluminescent labels and magnetic nanoparticles use DNA capture probes coupled to magnetic nanoparticles. By including a long spacer arm in magnetic nanoparticles, freedom for DNA hybridization and chemiluminescence detection is increased. An ultrasensitive method based on this system has been developed for the hepatitis B virus as a proof of concept [85], but it could be applied to other viruses.

### 3.6. Other Applications of Nanoparticles in Imaging Diagnostic Techniques

Other imaging techniques, if not exactly used to identify pathogen presence, can be used in evaluating tissue and organ functionality, and taking all the obtained information as a whole can help in the diagnosis of pathological conditions, as has been proved for liver imaging using SPION- enhanced magnetic resonance imaging (MRI) in hepatic cancer [122]. 

Considering the extended and consolidated application of gadolinium (Gd) as a contrast agent, Gd based nanoparticles have been explored. Different modifications of these nanoparticles with chelators, linkers, conjugation, coating and so on have been studied in order to enhance image contrast, which would lead to a better visualization of the diseases [15].

Some authors have suggested that virus-like particles can be used for encapsulation of contrast agents, improving their properties, as has been demonstrated for Fe_3_O_4_ nanoparticles encapsulated into hepatitis B core as a T2 MRI contrast imaging agent [80].

### 3.7. Virus-Based Nanoparticles in Viral Genetic Material Detection

Viral capsids have been used to shield nucleic acids increasing their stability during assays and storage, in order to be used as controls in diagnostic assays. Thus, the so-called “armored RNA” has been used for a variety of techniques to detect pathogens, including a vast variety of viruses [123]. Different modifications have been studied, for instance, to allow the simultaneous detection of several viruses. Following the same line, encapsulation of DNA in viral capsids has been also studied [15,124,125,126,127].

## 4. Bacterial Treatment: Quorum Sensing

**Figure 3:**
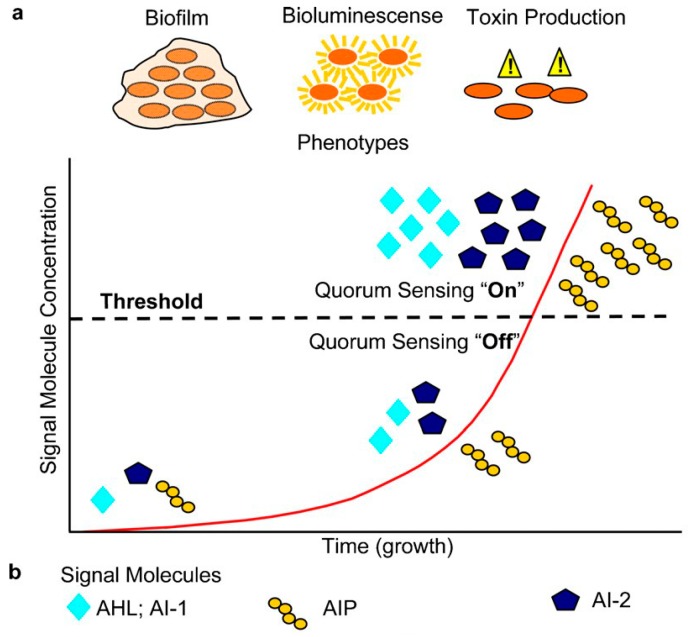
Schematic of the quorum sensing “Switch”, types of signal molecules and phenotypes. Autoinducers synthesis increases with bacteria population density (**b**) and when a thresthold level is achieved the response is activated (**a**) [134]. Reproduced with permission.

Bacteria are able to communicate with each other through secreted signaling factors, also named autoinducers. These chemical signals are synthesized intracellularly and released to the extracellular medium where they are recognized by the adjacent cells activating the expression of related genes [128]. The autoinducer activity and the behavioral changes are only triggered when a threshold level is reached [129,130,131,132,133]. These events require, then, high cell densities (to accumulate sufficient signal). The minimum behavioral unit has been described as a quorum of bacteria and thus, this mode of bacterial communication has been termed quorum sensing (QS) [134] as illustrated in Figure 3.

The QS process among cells was first discovered to control bioluminescence in the marine bacteria *Vibrio fischeri*, where for low cell densities an homoserine lactone is secreted to the medium, whereas for high cell densities it is accumulated inside when it triggers the transcription of luminescence genes [133,135]. It is now known that QS modulates many biological properties in bacteria as the production of virulence factors and the bacterial biofilms [136,137,138]. Thus, in *Pseudomonas aeruginosa*, whose quorum sensing system has been the most studied, it regulates the production of several compounds that play important roles in biofilm formation. This includes rhamnolipids, lectin A (LecA)/LecB and pyochelin and pyoverdine siderophores [132,139,140,141,142].

Hence, the functional criteria defining a QS system is: a cell density-dependent accumulation [143] of a small diffusible molecule that is recognized by adjacent cells, in which it triggers a specific transcriptional response [144].

Different QS pathways have been identified [130]. The quorum sensing systems can be classified into several groups, based on the type of auto-inducer used [129].

The QS system regulated by acyl-homoserinelactone (AHLs) is a standard mechanism most commonly employed by Gram-negative bacteria species. AHLs, also known as autoinducer-1 (AI-1), are molecules composed of a lactone ring and an aliphatic chain whose length and nature may vary for different species [145]. The LuxI/LuxR protein system controls this type of communication [146,147]. A LuxI protein synthesizes an acylated homoserine-lactone (AHL) using the substrate S-adenosylmethionine (SAM) and an acylated acyl-carrier protein (acyl-ACP) [148] AHL molecule, which starts the quorum sensing circuit, generally diffuses freely across the cell envelope and accumulates in the local environment [149]. When an adequate level of AHL is reached, it is recognized by a LuxR protein that results in the activation of related genes [129,146].

Other types of homoserine-lactone synthase, LuxM and AinS, have been discovered in vibrio species. Although they present important sequence differences with LuxI, their substrates and reaction kinetics are similar [128,150,151].

**Figure 4:**
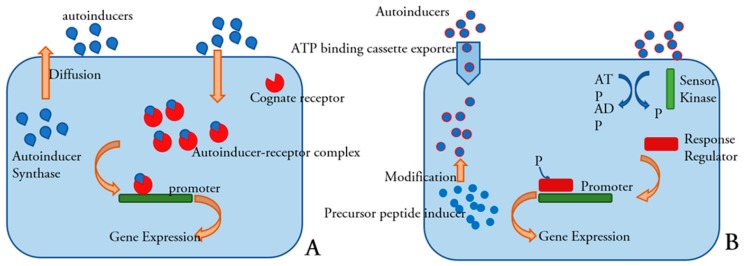
General scheme of quorum sensing in Gram-negative (**A**) and Gram-positive (**B**) bacteria [155]. Reproduced with permission.

Gram-positive bacteria generally employ for communication small peptidic molecules named auto-inducing peptides (AIP). The synthesis of these peptide signals occurs in the cell ribosome and they undergo some modifications to reach a matured form. Specialized transporters are responsible for its active transport out of the cell. Detection of these peptide signals can occur either at the surface of the cell or intracellularly [149]. The simplest Gram-positive quorum sensing system was first discovered in *Lactococcus lactis* and *Streptococcus pneumoniae* [152]. A precursor of the auto-inducing peptide synthesized in the cell ribosome is modified and processed by an ATP-binding cassette (ABC) complex and then transported out of the cell At a certain AIP concentration, a two-component regulatory system transduces the signal through a phosphorylation cascade to response regulators that activate or repress target genes (Figure 4) [129,149,150,152,153,154]. 

The third main category of autoinducers is AI-2, used by bacteria for interspecies communication. It is a furanosyl borate diester, produced by a LuxS protein [129,156]. It has been identified in over 60 bacterial species although only a few of them possess receptors that sense and respond to the presence of AI-2. This autoinducer regulates the production of virulence factors in *Vibrio vulnificus* [157,158,159], *Serratia marcescens* [159] and *Clostridium perfringens* [160]. However, the specific role of AI-2 in other species of bacteria remains unresolved [129,130].

Other molecules are also employed by intraspecies QS. This includes 2-heptyl-3hydroxy-4-quinolone, also referred to as *Pseudomonas*quinolone signal (PQS). In *Pseudomonas aeruginosa*, virulence factors such as pyocyanin, elastase and LecA lectin, which significantly impact the infection process are regulated by the PQS [149,161]. 

The diffusible signal factor (DSF) family are cis 2-unsaturated fatty acids that have been found in many bacterial species where they regulate motility, biofilm formation, iron uptake, extracellular polysaccharide (EPS), extracellular enzyme production and virulence [162]. It has also been proposed that they could play a role in interspecies communication [163]. These kinds of autoinducers were first described for *Xantomonas* spp. They have now also been found in *P. aeruginosa* and other species [162,164]. A signal turn over mechanism for DSF has been described [165,166].

Other QS systems found in various *Vibrio* spp. as well as in *Legionella* utilize α hidroxiketones (AHKs) as signaling molecules [149]. 

Furthermore, it is becoming evident that bacteria do not usually rely on only one signal molecule, and different QS systems with hierarchical or parallel performance have been described [167]. 

### 4.1. Quorum Sensing Interfering Agents: An Alternative to Antibiotics for Controlling Bacterial Infections

Nowadays, the increase in resistant bacterial strains and the lack of new antibiotics make it necessary to search for new strategies to fight infections. Due to the important role that quorum sensing plays in bacterial virulence, the disruption of this bacterial communication systems is attracting a great deal of interest as a new antimicrobial strategy [131,168]. These are very promising approaches because as they do not directly compromise bacterial survival, involve low selection pressure and thus yield a lower occurrence of resistance [130,145]. 

This intervention strategy is named “quorum quenching” (QQ), a term used to include any approach that interferes with proper microbial QS signaling [169,170,171]. This can be done at different points: inhibition of autoinducer synthesis, degradation of the autoinducer and interception of its interaction with the receptor [130,172]. 

Depending on the type of regulation (i.e., whether quorum sensing induces or represses virulence), the agents will need to either inhibit or stimulate quorum-sensing-regulated gene expression. The latter is the case for the human pathogen *Vibrio chlolerae*, in which quorum sensing represses biofilm formation and virulence factor production [130,168]. 

Many compounds have been described as inhibiting the bacterial communication process. Quorum sensing interfering (QSI) agents can be either natural or synthetic compounds, as well as small molecules, enzymes and antibodies. Excellent and complete reviews of QS inhibitors have been published [130,134,149,153,155,168,170,171].

There have been attempts to develop inhibitors of Lux synthases [132] like butyryl-SAM and sinefungin [173]. However, the most extensively studied QSI strategies to date are degradation and modification of the quorum sensing signals themselves and the use of autoinducer antagonists.

Most enzymes identified thus far target AHL, although enzymatic inactivation of other signals has also been reported. The main source of those enzymes are another bacterial, fungal, plant or animal species that secrete them as a defense mechanism against the QS that bacteria use to compete with them [174].

There are three known classes of enzymes which target AHL signals: lactonases, acylases and oxidorreductases. They are obtained from diverse bacterial species. 

The lactonases belong to the metallo β lactamase superfamily. They inactivate AHL by hydrolyzing the ester bond, causing the opening of the homoserinelactone ring Acylases break the AHL amide, rendering the fatty acid and the homoserinelactone ring, products that do not show any activity [174,175]. As the AHL of different bacterial species maintain the homoserinelactone ring, lactonases are active for all of them [176,177]. Acylase activity depends on AHL fatty acid substitutes or chain length, which show variations between species [178,179,180]. Oxidoreductases are the most recently discovered AHL enzymes inactivating group. They modify but do not break AHL, transforming oxo-AHL into the corresponding hidroxyAHL. This can affect AHL receptor binding [181,182]. 

QQ enzymes can also be found in animals. This is the case of mammalian paraoxonases, a group of esterases present in sera and other tissues. They have shown hydrolytic activity for *N*-(3-Oxododecanoyl)-l-homoserine lactone (3OC12-HSL) of *Pseudomonas aeruginosa* [183,184,185]

The use of autoinducer antagonists is another strategy used for inhibiting the QS. Many compounds that are structurally similar to those of quorum sensing signal molecules have also been identified [155,171]. The best studied QSI with this model of action are halogenated furanones, a group of compounds initially obtained from red algae. These compounds can disrupt AHL signaling pathways by reducing the affinity of the autoinducer to the Lux-R or by interfering with the signal transduction. Also, some furanones are able to inhibit the synthase LuxS, the enzyme responsible for AI-2 [168,186,187]. 

Many medicinal plants, species such as garlic, ginger, essential oils of cinnamon and clove are also known to possess QSI activities. Carrot, chamomile, garlic and many peppers have been proven to have anti-QS activity, although the mechanisms for many of them have not yet been identified. Also, flavonoids such as baicalin, quercetin, naringenin, kempferol, apigenin have all been reported to be efficient in antagonizing bacterial QS [155].

The question of whether anti-QS therapies should be used as postinfection interventions or as prophylactic measures remains to be solved. In most studies, a prophylactic use has been studied by administering the anti-QS agents at the same time as the pathogens inoculum and a profound improvement in the infection outcome has been found [188,189,190]. Therapeutic benefits of QSI have also been obtained against microorganisms such as *Salmonella typhimurium* and *Serratia marcescens* when administering the anti-QS after the infection has developed [191,192]. This supports the use of these products as new antibiotics.

### 4.2. Nanoparticles and Quorum Sense Inhibitors

Many nanoparticle systems have been proposed for biofilm control but here we are only going over studies that correlate these systems with quorum sensing inhibition. Thus, we are focusing on the research about the properties of nanomaterials as QS inhibitors as well as the use of these systems as carriers of molecules with quorum quenching activity.

The quorum quenching abilities of metals and metallic nanoparticles have been especially highlighted [193]. The anti-QS activity of silver NPs has been the most widely studied among them Silver nanoparticles showed a reduction of AHL bacterial synthesis in two soil *Pseudomonas* species at sub-inhibitory concentrations. However, a species-dependence activity was observed [194]. In another study, silver NPs reduced the levels of AHL and its transcriptional regulators LasIR and RhIiR in *Pseudomonas aeruginosa*, inhibiting biofilm formation and the production of virulence factors such as LasA protease, LasB elastase, pyocianin, pyoverdin, pyochelin, rhamnolipid and alginate [195]. Ant-iQS activity of silver NPs has also been assessed for uropathogens *S. marcescens* and *Escherichia coli* resulting in the inhibition of QS virulence factors such a as prodigiosin and protease [192].

When AgCl was incorporated to TiO_2_ NPs (ATNPs), it also showed anti-QS activity against the biosensor *Chromobacterium violaceum*, a Gram negative bacteria with acyl homoserine lactone autoinducers. The anti-QS activity of ATNPs was achieved at a concentration that was 20 times lower than the bactericidal concentration. TiO_2_ acted as a good supporting matrix, facilitating effective use of silver by reducing the concentration required for bioactivity [196]. This strategy has been proposed for packaging materials in order to prolong food shelf-life.

ZnO nanoparticles have also shown antibiofilm activity against *Pseudomonas aeruginosa* at concentrations below its bactericidal level. The mechanism involves quorum sensing processes that lead to an inhibition of biofilm formation and pyocyanin, pseudomonas quinolone signal (PQS) and pyochelin production [197].

β-cyclodextrins are cyclic oligosaccharides used as excipients for the improvement of water-solubility and bioavailability of medicinal products. They have been shown to non-specifically bind to AHL, thus removing these signal molecules from the bacterial environment and disrupting bacterial QS. Its fixation to the surface of silicon dioxide nanoparticles increases its quenching ability [198].

Plain nanocapsules of another polysaccharide, chitosan, which contained an oil core stabilized by a surfactant and coated by the polymer, also inhibited the quorum sensing of *Escherichia coli* [199]. In another study chitosan/pentasodium tripolyphosphate (TPP) NPs loaded with kaempherol showed anti QS activity mediated by AHL as established using the biosensor *Chromobacterium violaceum*. The encapsulated flavonoid could act as a stronger and longer acting quorum quencher than kaempherol alone [200]. The encapsulation of other flavonoids, quercetin and baicalein, in chitosan nanocapsules also improved its antiquorum sensing activity in comparison with the free form. The anti-QS activity was measured in the *E. coli* Top 10 biosensor and the mechanisms proposed were inhibition of the autoinducer synthase, (a modification of LuxR receptor) that could affect its union with the DNA promoter, or the blockage of the diffusion of AHL to the cytosol [201].

The insolubility of many molecules that are inhibitors of QS limit their potential to be used as drugs [131]. Recently, a PqsR antagonist, thus linked to the PQS signal: 2-heptyl-6-nitro-4-oxo-1,4-dihydroquinoline-3-carboxamide, was described as strongly inhibiting the virulence of *Pseudomonas aeruginosa*. As it is a very lipophilic molecule, it was formulated in solid lipid nanoparticles (SLN) and the anti-QS activity was evaluated. It was found that the loaded NPs were superior to the free QSI in the inhibition of pyocianin formation. Also, a strong inhibition of this virulence factor was achieved with plain SLN. This effect was not due to the killing of bacteria and was attributed to the emulsifiers present in the formulation, mainly Tween 80 and Poloxamer 407 [202].

The highly hydrophobic molecule (cLogPof 4.6) *Vibrio chlolerae* quorum-sensing signal CAI-1 (*S*-3-hidroxytridecan-4-one) is a promising candidate for a quorum sensing agonist whose accumulation induces transition of *V. chlolerae* to a non-biofilm forming non-virulence state. The process, called “Flash-nanoprecipitation”, was used to incorporate this compound into nanoparticles of polystyrene-block polyethylene glycol (PEG) and vitamin E to create a water dispersible form intended for oral delivery. These particles activated *V. chlolerae* quorum-sensing responses five orders of magnitude higher than the identically administered free CAI-1 does, and were able to diffuse across murine small intestinal mucus [203].

The combination of antibiotics and QSI might offer significant synergy such as reducing bacterial virulence factor expression and thus protecting the host and making pathogens more susceptible to immune defense [204]. Likewise, co-encapsulation of other antimicrobial substances together with an antibiotic into liposomes could improve antimicrobial efficacy. The antibacterial and antibiofilm activity of metals has supported its incorporation into these combinations. Thus, the coencapsulation of gallium with gentamycin in dipalmitoyl phosphatidylcholine:dimirystoil phosphatidylcholine:cholesterol (DPPC:DMPG:Chol) liposomes (Lipo-Ga Gen) showed better antibiofilm activity against *Pseudomonas aeruginosa* than the free combination or each compound separately. Only liposome formulation was able to suppress AHL production [205]. Other studies with liposomes combining bismuth–ethanedithiol with tobramycin also showed the superior ability of liposomes inhibiting the production of *N*-acyl homoserine lactone, and virulence factors elastase, protease and chitinase [205,206,207,208,209,210]. 

Also, several niosome formulations, including tobramycin and bismuth-ethanedithiol, were evaluated both alone and in combination as drug delivery systems with regards to quorum sensing and biofilm formation in various strains of *Pseudomonas aeruginosa*. The composition of niosomes included: Span 40, Tween 40 and 30% cholesterol. The incorporation in niosomes reduced the minimum inhibitory concentration (MIC) of formulations including tobramycin in comparison with the non-encapsulated ones. This has been attributed to an enhanced penetration of the antibiotic in the cell [210]. Furthermore, 1:2 MIC and 1:4 MIC concentrations of the free and niosomal bismuth-ethanedithiol tobramycin combinations were the most effective, inhibiting biofilm formation by over 80%. These two formulations were also able to inhibit AHL production [211].

Despite the huge number of molecules described as quorum sensing inhibitors, as well as the massive research about nanoparticles used for infections and biofilms treatments, not many studies can be found about using nanoparticles in quorum quenching. However, nanoparticles as carriers of QSI can overcome the unfavorable properties of many of these compounds. Furthermore, besides the advantages of these systems based on their size, nanomaterials used may also have anti-QS activity. The metallic NPs show promising results in QS inhibition but also other compounds as tensioactives might have this activity. Further research in this interesting field must be done.

## 5. Conclusions

Numerous advances in nanoparticle-based systems for the diagnostic and treatment of bacterial infections have been recently published with potential applications in the fight against multidrug resistant strains and bacterial biofilms, among other areas. Those nanosystems may also be useful for diagnoses and treatment of viral infections.

The characteristics of certain types of nanoparticles and additional functionalization confer on them ideal properties for application in diagnostic analysis, even allowing miniaturization and simplification of some traditional techniques of pathogen detection. Advanced analytical methods, such as SERS, combined with the use of metallic nanoparticles are excellent tools for the detection of bacteria and viruses. Also, other methods, such as plasmonic nanocavities or magnetic and electrochemical biosensors, have been implemented. As a result, more sensitive and easy to use techniques have been achieved. Moreover, nanoparticles constitute an interesting platform for theranostic applications.

In addition, the inhibition of bacterial quorum sensing systems by the use of metallic and other types of nanoparticles constitutes a promising strategy in the fight against bacterial infections. The incorporation of QS inhibitors into these nanosystems increases their efficacy for biofilm treatment.
